# Popular Science Monthly

**Published:** 1879-01

**Authors:** 


THE POPULAR SCIENCE MONTHLY.—Contains monthly articles, on by far the most interesting topics known to scientific men, and we beg to call attention to the following notable articles in *The Popular Science Monthly Supplement* for December: Recent developments of Socialism in Germany and the United States. The Alcohol Question. A Symposium by three of England's most noted Physicians. Malt-Liquors—Their effect on Digestion and Nutrition. American Facts and Gladstone Fallacies. Outside of our own professional journals no publication interests us so much as this. Every Physician should read it.

